# A rising tide lifts all MBOATs: recent progress in structural and functional understanding of membrane bound *O*-acyltransferases

**DOI:** 10.3389/fphys.2023.1167873

**Published:** 2023-05-04

**Authors:** Mariah R. Pierce, James L. Hougland

**Affiliations:** ^1^ Department of Chemistry, Syracuse University, Syracuse, NY, United States; ^2^ Department of Biology, Syracuse University, Syracuse, NY, United States; ^3^ BioInspired Syracuse, Syracuse University, Syracuse, NY, United States

**Keywords:** membrane-bound *O*-acyltransferase, cryoelectron microscopy, acylation, MBOAT fold, computational structure prediction, ghrelin, Wnt, Hedgehog

## Abstract

Acylation modifications play a central role in biological and physiological processes. Across a range of biomolecules from phospholipids to triglycerides to proteins, introduction of a hydrophobic acyl chain can dramatically alter the biological function and cellular localization of these substrates. Amongst the enzymes catalyzing these modifications, the membrane bound *O*-acyltransferase (MBOAT) family occupies an intriguing position as the combined substrate selectivities of the various family members span all three classes of these biomolecules. MBOAT-dependent substrates are linked to a wide range of health conditions including metabolic disease, cancer, and neurodegenerative disease. Like many integral membrane proteins, these enzymes have presented challenges to investigation due to their intractability to solubilization and purification. However, over the last several years new solubilization approaches coupled with computational modeling, crystallography, and cryoelectron microscopy have brought an explosion of structural information for multiple MBOAT family members. These studies enable comparison of MBOAT structure and function across members catalyzing modifications of all three substrate classes, revealing both conserved features amongst all MBOATs and distinct architectural features that correlate with different acylation substrates ranging from lipids to proteins. We discuss the methods that led to this renaissance of MBOAT structural investigations, our new understanding of MBOAT structure and implications for catalytic function, and the potential impact of these studies for development of new therapeutics targeting MBOAT-dependent physiological processes.

## 1 Introduction

### 1.1 Membrane bound *O*-acyltransferase family—history and background

The membrane bound *O-*acyltransferase (MBOAT) family comprises a group of enzymes characterized by multiple transmembrane domains and a conserved histidine residue. The first family member reported in the literature was acyl-coenzyme A: cholesterol acyltransferase, also known as sterol *O-*acyltransferase (ACAT1/SOAT1) (Mukherjee and Alfin-Slater, 1958; Goodman et al., 1964). ACAT1/SOAT1 is responsible for the acylation of the alcohol on cholesterol to form cholesterol esters. ACAT1/SOAT1 was first extracted from rat liver homogenates and was later identified as a membrane bound enzyme (Mukherjee and Alfin-Slater, 1958; Goodman et al., 1964). Identification of the gene encoding ACAT1/SOAT1, the *ACAT/SOAT* gene, led to discovery of the homologous ACAT2/SOAT2 (Chang et al., 1993; Yang et al., 1996; Yu et al., 1996; Cases et al., 1998a; Anderson et al., 1998; Oelkers et al., 1998). In addition to ACAT2/SOAT2, a related enzyme diacylglycerol *O*-acyltransferase 1 (DGAT1) was identified by sequence similarity to ACAT1/SOAT1 (Cases et al., 1998b). This enzyme performs similar acylation modifications on a distinct substrate diacylglycerol with involvement in triglyceride biosynthesis. Further sequence analysis led to yet another enzyme, Porcupine (PORCN) (Hofmann, 2000). PORCN is an acyltransferase in the Wnt signaling pathway, where it acylates the secreted signaling protein Wnt in contrast to the lipid and cholesterol substrates for ACAT1/SOAT1 and DGAT1. In 2000, Hofmann identified PORCN as an additional acyltransferase and named this enzyme family the membrane bound *O*-acyltransferases (MBOAT) (Hofmann, 2000).

### 1.2 MBOAT family: acylation substrates lead to subfamily classifications

**FIGURE 1:**
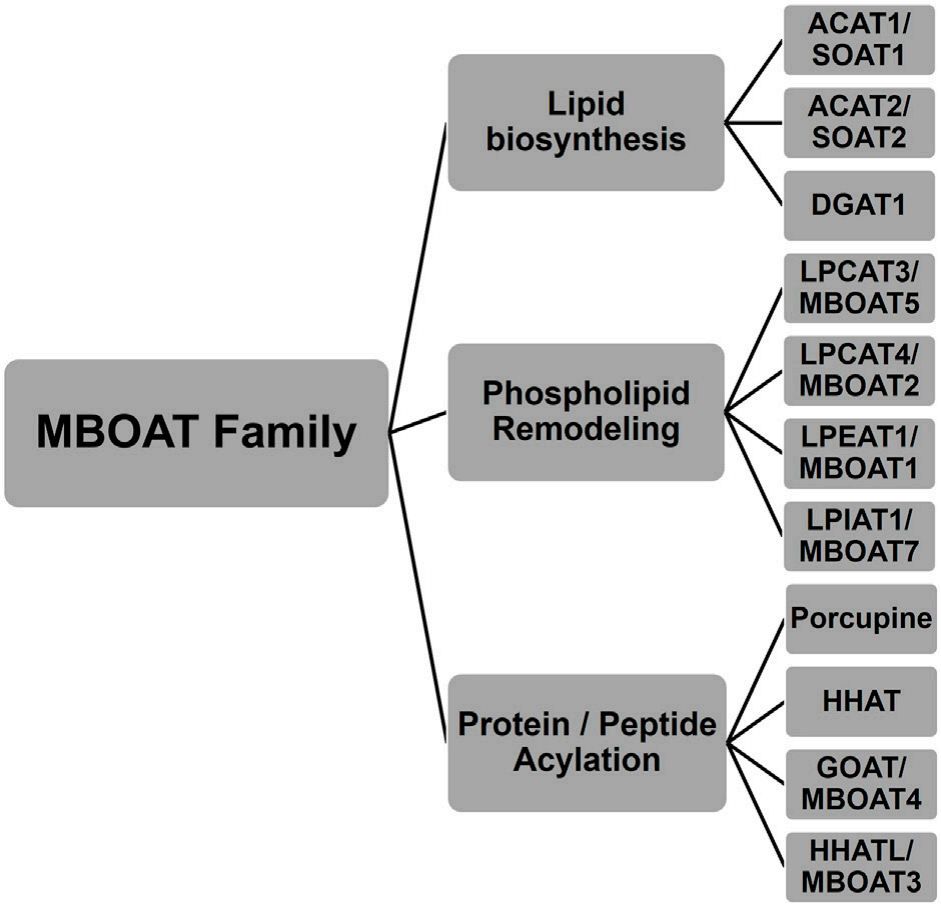
Membrane bound *O*-acyltransferase (MBOAT) family of enzymes. These integral membrane enzymes acylate cholesterol, diacylglycerol, phospholipids, peptides, and proteins.

The MBOAT family of enzymes can be classified by the biochemical reaction each enzyme performs (Figure 1). MBOAT enzymes were first classified as enzymes that perform lipid biosynthesis. This group includes the ACAT/SOAT enzymes and DGAT1 that acylates cholesterol and triglycerides. Another group of MBOATs are lysophospholipid acyltransferases responsible for acylating phospholipids. The last group is responsible for acylating proteins and peptides. Below we describe the known mammalian MBOATs and their major roles in the cell. In section c we will describe their roles in signaling and disease.

#### 1.2.1 Lipid Biosynthesis

As noted above, ACAT1/SOAT1 is responsible for the acylation of cholesterol to cholesterol ester. Cholesterol esters provide a storage option to prevent cholesterol build-up in cell membranes. Consequently, ACAT1/SOAT1 is expressed in multiple cell types in the body. Chang and co-workers expressed and purified ACAT1/SOAT1 with full biological activity in 1998 (Chang et al., 1998). ACAT1/SOAT1 is a homotetrameric enzyme with 9 transmembrane domains per monomer, with this multimeric nature posing difficulties for enzyme studies (Yu et al., 1999). Two conserved amino acids, His460 and Asn421, have been implicated in its acylation activity (Guo et al., 2007). ACAT1/SOAT1 will bind sterols and steroids and contains multiple binding sites for these substrates.

ACAT2/SOAT2 is mainly found in the small intestine and liver (Anderson et al., 1998). Its expression and cloning was also reported in 1998 (Cases et al., 1998a). The predominant theory for the existence of both enzymes is that ACAT1/SOAT1 acts to maintain cholesterol levels throughout the body whereas ACAT2/SOAT2 is coupled to lipoprotein particle assembly and secretion (Joyce et al., 2000). Further analysis of acylation by these enzymes has been pursued using the structure publication that will be described below.

While ACAT/SOAT modifies cholesterol, acyl-CoA: diacylglycerol acyltransferase (DGAT) acylates precursors to create triglycerides. Triglycerides are used for energy storage and membrane lipid formation. While triglycerides are important for normal physiological behavior, an excess of these glycerol triesters can lead to disease states such as obesity (Birch et al., 2010; Lee et al., 2010). In 1956, it was reported that DGAT used fatty acyl-CoAs as acyl donors (Weiss and Kennedy, 1956), and the connection to the ACAT enzymes at the sequence level came in 1998 (Weiss et al., 1960; Cases et al., 1998b). Knockout of both DGAT1 and DGAT2 in mice lead to reduced triacylglyceride levels (Smith et al., 2000; Stone et al., 2004). DGAT1 prefers monosaturated substrates where DGAT2 did not show a saturation preference but a chain length preference. DGAT2 prefers shorter chain acyl-CoAs and short/medium chain fatty acyl moieties (Cases et al., 2001; Lardizabal et al., 2001). DGAT2 is more efficient at triacylglycerol (TAG) acylation, while DGAT1 has the potential to acylate multiple substrates (Ross, 1982; Batten et al., 2004; Yen et al., 2005). This is further believed to be true as their topologies are drastically different with DGAT1 containing multiple transmembrane domains and DGAT2 with significantly fewer domains (Cases et al., 2001; Cheng et al., 2001; Lardizabal et al., 2001; Weselake et al., 2006). For more detailed discussion of DGAT1 and DGAT2 activity, expression, and topology, the authors direct to the following excellent reviews on these topics by Yen et al., in 2008 and most recently by Chen et al., in 2022 (Yen et al., 2008; Chen et al., 2022).

#### 1.2.2 Lysophospholipid acyltransferases

**FIGURE 2:**
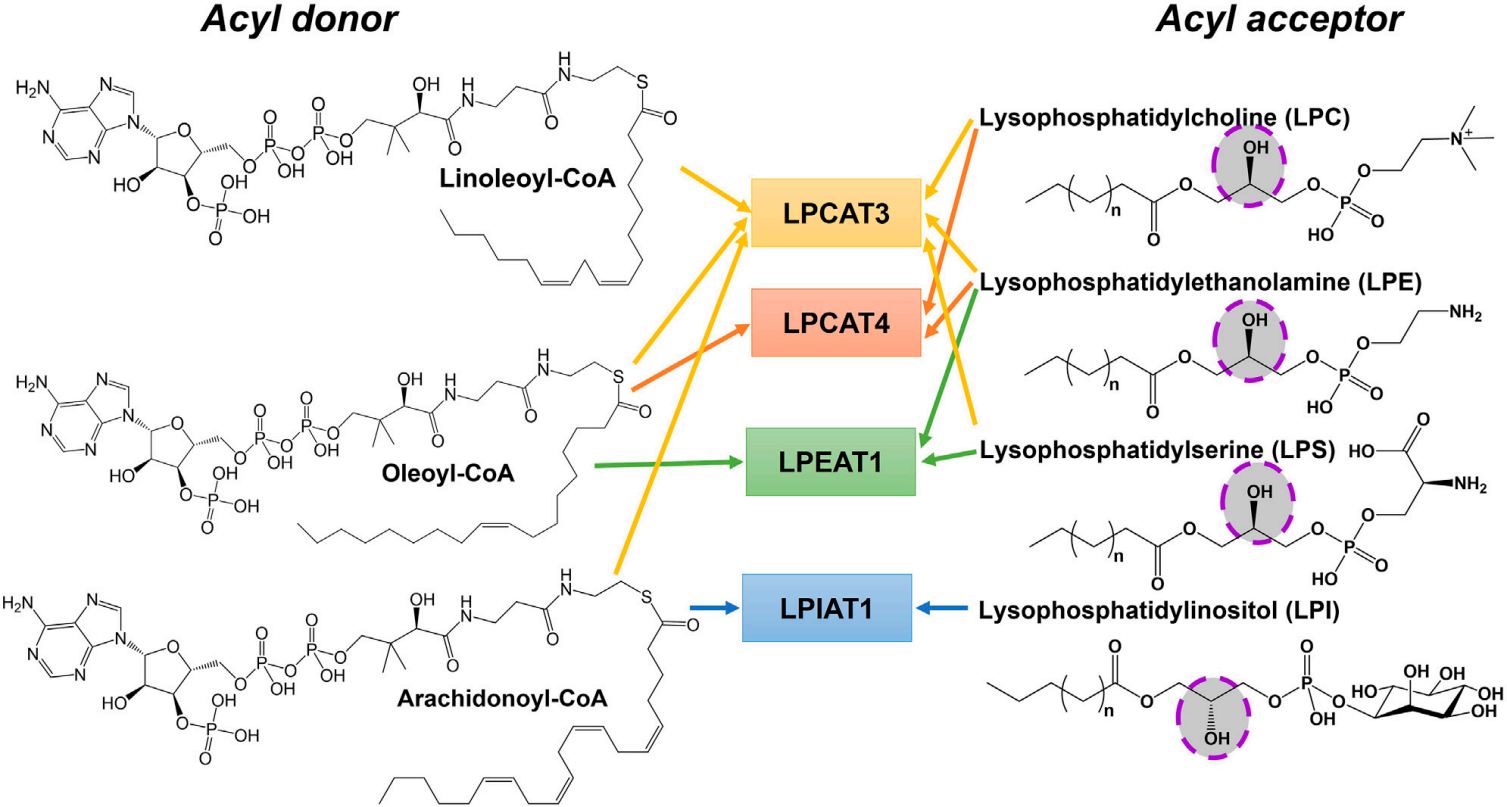
Phospholipid remodeling MBOAT enzymes have multiple substrates and utilize multiple acyl-CoAs as acyl donors. Phospholipid remodeling enzymes can acylate various substrates using a range of acyl donors. This figure shows a subset of these potential enzyme-acyl acceptor–acyl donor combinations for acyl donors bearing unsaturated fatty acids. The hydroxyl group that becomes acylated in the acyl acceptor substrate is highlighted.

MBOAT family members also play key roles in phospholipid synthesis and recycling (Figure 2). Phosphatidylcholine (PC), phosphatidylethanolamine (PE), and phosphatidylserine (PS) are the major phospholipids in membranes and surfactants in mammals. PC acyl chain remodeling is performed by lysophosphatidylcholine acyltransferases (LPCATs). Only LPCAT3 and LPCAT4 acylate lyso-PC with unsaturated acyl chains at the *sn-2* hydroxyl, with both of these enzymes annotated as MBOAT family members (Hofmann, 2000; Hishikawa et al., 2008). LPCAT3/MBOAT5 has seven transmembrane domains and the conserved His and Asn residues that typify MBOAT family members. LPCAT3/MBOAT5 favors unsaturated fatty acyl-CoAs such as oleoyl-CoA, linoleoyl-CoA, and arachidonyl-CoA as acyl donors and 1-myristoyl-lyso-PC and 1-palmitoyl-lyso-PC as acyl acceptors/acylation substrates (Zhao et al., 2008; Jain et al., 2009). LPCAT4/MBOAT2 and LPEAT1/MBOAT1 both prefer oleoyl-CoA as their acyl donor and catalyze reactions with lyso-PC, lyso-PE, and lyso-PS (Hishikawa et al., 2008). The last lysophospholipid acyltransferase amongst the MBOATs is lysophosphatidylinositol acyltransferase 1 (LPIAT1). LPIAT1/MBOAT7 adds an arachidonic acid or an eicosapentaenoic acid onto lyso-PI (Caddeo et al., 2019; Caddeo et al., 2021). LPIAT1 catalyzes the transfer of an acyl chain to lyso-PI and mutations to its conserved Asn321 and His356 resulted in a loss of acyltransferase activity like most other MBOAT family members (Caddeo et al., 2021). LPIAT1 prefers polyunsaturated fatty acids as opposed to saturated or unsaturated fatty acids (Caddeo et al., 2021). LPIAT1 has a predicted six transmembrane domains and is an integral membrane enzyme tightly bound to endosomes (Caddeo et al., 2019).

#### 1.2.3 Acylation of proteins/peptides

**FIGURE 3:**
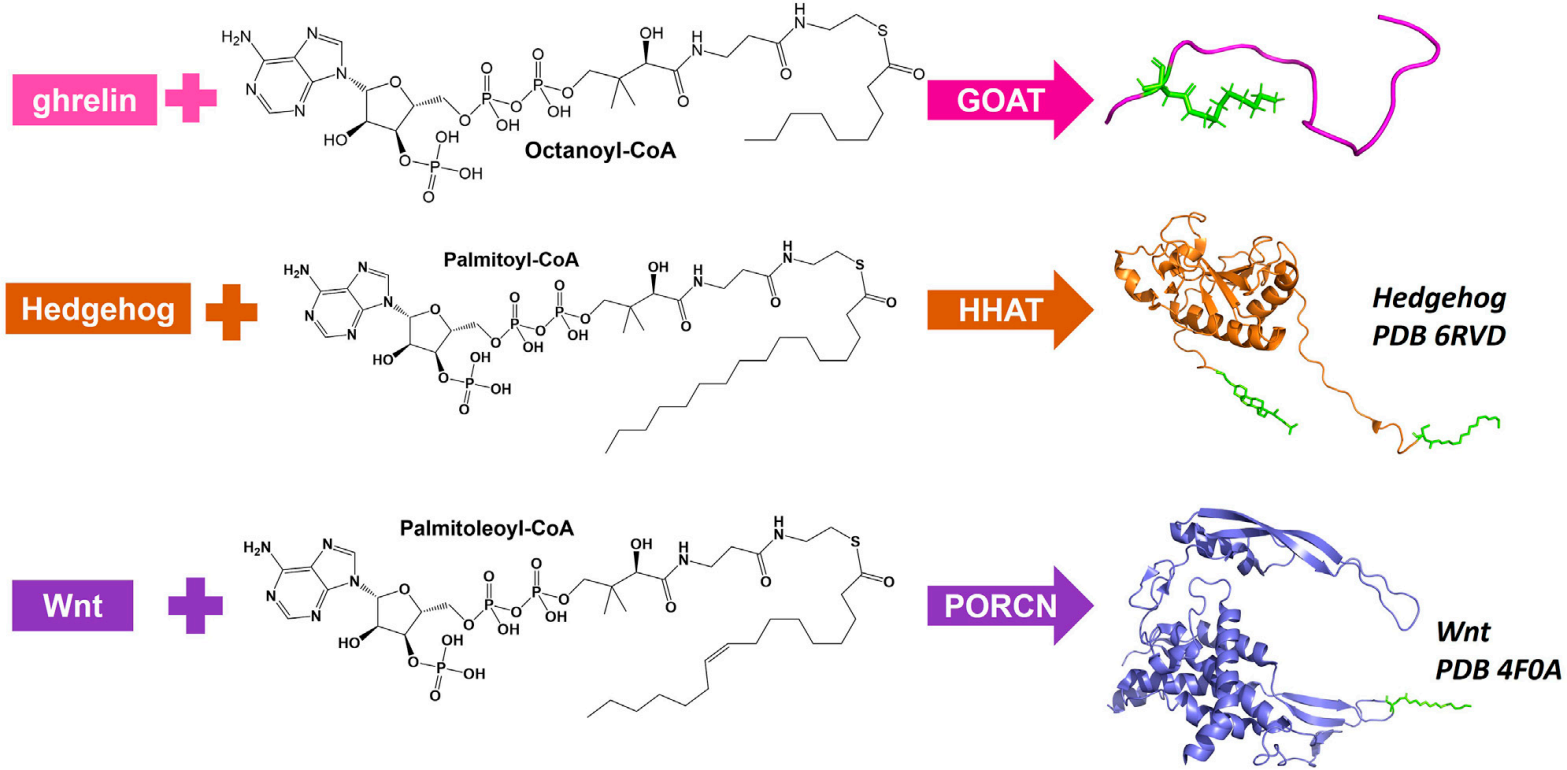
Protein and peptide acylating MBOATs. GOAT acylates the peptide hormone ghrelin and prefers octanoyl-CoA as the acyl donor. Hedgehog (PBD: 6RVD) first gets modified with a cholesterol moiety (green) then gets acylated by HHAT with a preference for palmitoyl-CoA. Wnt (PDB: 4F0A) is acylated by PORCN using palmitoleoyl-CoA.

There are three MBOATs that catalyze the acylation of proteins or peptides, PORCN, Hedgehog acyltransferase (HHAT), and ghrelin *O*-acyltransferase (GOAT/MBOAT4) (Figure 3). PORCN, identified as the first protein acylating MBOAT, acylates the signaling protein Wnt with a palmitoleate (C16:1) group (Zhai et al., 2004; Rios-Esteves et al., 2014). This modification occurs at a conserved serine and is required for receptor (Frizzled) binding and signaling (Takada et al., 2006; Janda et al., 2012). HHAT catalyzes the acylation of Hedgehog proteins (Sonic, Indian, and Desert) which bind and signal through the Patched receptor(Kong et al., 2019). HHAT palmitoylates (C16:0) the N-terminal cysteine on SHH (Pepinsky et al., 1998). GOAT octanoylates ghrelin on the third serine, and like HHAT/hedgehog and PORCN/Wnt this acylation modification is required for ghrelin binding and signaling through its receptor GHS-R1a (Kojima et al., 1999; Abizaid and Hougland, 2020).

### 1.3 Importance of MBOATs in signaling and disease

#### 1.3.1 ACAT/SOAT

Multiple studies show ACAT/SOAT to be an important enzyme to facilitate improvement in human health and disease. For example, inhibition of ACAT1/SOAT1 was shown to lower the level of neurological disease in an Alzheimer’s mouse model (Hutter-Paier et al., 2004; Bhattacharyya and Kovacs, 2010; Bryleva et al., 2010). It has been shown that by inhibiting ACAT1/SOAT1 cholesterol can be re-directed to repair other organelles (Hutter-Paier et al., 2004; Bhattacharyya and Kovacs, 2010; Bryleva et al., 2010). By way of modulating T-cell cholesterol metabolism, ACAT1/SOAT1 has been shown to facilitate cancer treatment (Yang et al., 2016). ACAT2/SOAT2 is only active in the hepatocytes and intestinal cells, and when ACAT2/SOAT2 is not functioning normally it can cause reduced assembly and secretion of low-density lipoprotein (LDL). This eliminates hypercholesterolemia and atherosclerosis (Buhman et al., 2000; Willner et al., 2003; Rudel et al., 2005; Ohshiro et al., 2015). Related to those diseases, a buildup of cholesterol and lipids in circulation can lead to heart attacks. Inhibiting ACAT2/SOAT2 has been suggested to be especially helpful in preventing these cardiovascular risks (Goldstein and Brown, 2009).

#### 1.3.2 DGAT1

DGAT1 is expressed in multiple tissues throughout the body. It has a major role in dietary fat absorption and protecting the body by preventing fatty acid (FA)-induced toxicity (Yen et al., 2008). Unesterified FAs promotes endoplasmic reticulum (ER) stress leading insulin resistance and impaired (Chitraju et al., 2017). DGAT1 performs the last step in TAG biosynthesis in the Kennedy and monoacylglycerol (MG) pathways, and without DGAT1 there was a severe loss in dietary fat absorption (Buhman et al., 2002). Inhibition of DGAT1 would prevent the absorption of TAG into the body, leading to reduced lipid storage in the body (Smith et al., 2000; Chen et al., 2002). Consequently, DGAT is a target for prevention of obesity, glucose metabolism, and insulin secretion.

#### 1.3.3 LPCAT3/MBOAT5

LPCAT3/MBOAT5 catalyzes a polyunsaturated acyl moiety (18:2 and 20:4) onto lyso-PC, lyso-PE, and lyso-PS (Hishikawa et al., 2008). This changes the composition of the cell membrane, and the function of the proteins in the surrounding area. LPCAT3 is a key component of the Kennedy pathway and the Lands’ Cycle (Kennedy and Weiss, 1956; Lands, 1958; Lands, 2000). LPCAT3 is primarily expressed in liver and is a key regulator of in phospholipid and triglyceride metabolism (Zhao et al., 2008). Of the four LPCATs, LPCAT3 is the major isoform in metabolic issues and has been proposed as a drug target for atherosclerosis and hyperlipidemia (Wang and Tontonoz, 2019; Liu et al., 2020). However, this approach has the potential for off target effects on cholesterol biosynthesis and fat accumulation (Rong et al., 2015; Wang et al., 2018). LPCAT3 has been implicated in obesity induced skeletal myopathy, with mice overexpressing LPCAT3 exhibiting worse skeletal myopathy when fed a high-fat diet then those mice fed a normal diet. LPCAT3 is consequently a therapeutic target for treatment of obesity induced skeletal myopathy (Zhang et al., 2012). LPCAT3 also plays a role in diabetes. Mice overexpressing LPCAT3 showed improved postprandial hyperglycemia and glucose tolerance (Ferrara et al., 2021). However, in skeletal muscle the opposite is observed with overexpression of LPCAT3 in skeletal muscle leading to glucose intolerance (Labonté et al., 2006). For more detailed discussion of recent progress in understanding LPCAT3’s role in cancer and diseases, the authors direct your attention to a recent review by Shao et al. (Shao et al., 2022).

#### 1.3.4 LPCAT4/MBOAT2

Like LPCAT3, LPCAT4 is also a key component of the Kennedy pathway and Lands’ Cycle (Kennedy and Weiss, 1956; Lands, 1958; Lands, 2000). LPCAT4 is also referred to acyl-CoA:lyso-PE (LPEAT2) due to its lyso-PE activity, but following the publication by Hishikawa and co-workers it is now only called LPCAT4 (Hishikawa et al., 2008). LPCAT4 is responsible for acylating lyso- PC and lyso- PE that make up the cell membrane (Hishikawa et al., 2008). LPCAT4 is expressed in the brain, testis, epididymis, and ovary (Cao et al., 2008; Hishikawa et al., 2008). LPCAT4’s expression is upregulated in colorectal cancer cells. The ratio of PC to Lyso-PC has been implicated as a biomarker for colorectal cancer, indicating LPCAT4 as a key factor for improving standard of care for colorectal cancer (Kurabe et al., 2013). LPCAT4 is known to regulate chondrogenic differentiation in skeletal development (Tabe et al., 2017). Suppressing LPCAT4 activity has recently been implicated to slow down pancreatic tumor progression (Zhou et al., 2021; Li et al., 2022; Xie et al., 2022).

#### 1.3.5 LPEAT1/MBOAT1

Lysophosphatidylethanolamine acyltransferase 1 (LPEAT1) acylates lyso-PE and lyso-PS with oleoyl-CoA (18:1) (Hishikawa et al., 2008). LPEAT1 is also a contributor to the Lands Cycle and Kennedy pathway (Kennedy and Weiss, 1956; Lands, 1958; Lands, 2000). The gene for LPEAT1 is located in chromosome 6 and when it is disrupted leads to brachydactyly-syndactyly syndrome (Dauwerse et al., 2007). Tabe et al. found that when LPEAT1 expression is knocked down, the growth of neurites decreased leading the authors to conclude that LPEAT is implicated in neurite outgrowth and function (Tabe et al., 2016). An exome screen in infertile Chinese male patients revealed two mtations in *MBOAT1,* the gene that encodes LPEAT1. This mutation, Thr257Met, impedes the translation of *MBOAT1* and leads to a lower expression of LPEAT1 (Wan et al., 2022). Similar evidence of infertility related to low LPEAT1 expression has been reported in *Drosophila* (Steinhauer et al., 2009).These authors are excited to see more developments from those labs studying LPEAT1.

#### 1.3.6 LPIAT1/MBOAT7

LPIAT1 is also one of the many acyltransferases in the Lands cycle (Lands, 1958; Lands, 2000). In a global study of patients with neurodevelopmental disorders, a significant number of patients had biallelic or pathogenic variants in LPIAT1 or MBOAT7 (Johansen et al., 2016). LPIAT1 was also shown to be required for correct brain development in mice (Lee et al., 2012; Anderson et al., 2013). Loss of lyso-PI acylation due to LPIAT1/MBOAT7 depletion resulted in a large increase in triglycerides in hepatocytes (Tanaka et al., 2021). Recently it has been suggested that LPIAT1 is a major contributor to liver disease, with a loss-of-function variant near *MBOAT7* gene associated with various liver diseases such as metabolic-associated fatty liver disease, nonalcoholic fatty liver disease, and alcohol-associated liver disease (Varadharajan et al., 2022).

#### 1.3.7 HHAT

Hedgehog signaling ligands were first discovered in *Drosophila* patterning (Nüsslein-Volhard and Wieschaus, 1980). Hedgehog acyltransferase (HHAT) catalyzes the lipidation of Hedgehog proteins (Buglino and Resh, 2008). This lipidation is essential to maintain Hedgehog signaling. Mutations to Hedgehog signaling proteins can cause congenital diseases and holoprosencephaly (Roessler et al., 1997; Briscoe and Thérond, 2013). Abnormal signaling of this pathway in involved in various malignancies including pancreatic, breast, and lung cancer (Konitsiotis et al., 2014; Wu et al., 2017; Chahal et al., 2018). The acylation activity of HHAT has been directly linked to pancreatic dual adenocarcinoma (Petrova et al., 2015). Hedgehog proteins are specific substrates for HHAT and this specificity makes HHAT a favorable pharmaceutical target. Abnormal Hedgehog signaling can be blocked by HHAT inhibitors. Multiple inhibitors have been designed to inhibit HHAT acylation activity, with photochemical probes utilized to identify the small molecule inhibitor binding site within HHAT (Lanyon-Hogg et al., 2015a; Lanyon-Hogg et al., 2015b; Lanyon-Hogg et al., 2016; Rodgers et al., 2016; Lanyon-Hogg et al., 2019; Lanyon-Hogg et al., 2021). One such example is RU-SKI 43 which was able to inhibit HHAT function *in vitro* and in cells with an IC_50_ of 850 nM (Petrova et al., 2013). A subsequent study demonstrated off-target cytotoxicity from RU-SKI 43 in cell studies and showed that a related compound RU-SKI 201 specifically inhibits HHAT acylation activity within cells with potencies in the range of 730–870 nM in independent assays (Rodgers et al., 2016).

#### 1.3.8 PORCN

For Wnt to be trafficked from the ER to the Golgi and bind to its subsequent receptor Frizzled, it must undergo palmitoleoylation by PORCN (Takada et al., 2006; Galli et al., 2016). This chemical modification is required for proper Wnt signaling. Consequently, PORCN has been implicated as an important target for inhibition in the Wnt pathway (Chen et al., 2009; Dodge et al., 2012). Wnt signaling is implicated in several cancers and orphan diseases. Inhibition of PORCN was shown to prevent the growth of mammary tumors in mice with little toxicity to the mouse (Proffitt et al., 2013). Another PORCN inhibitor, LGK974, was found to prevent Wnt signaling in murine and rat mechanistic breast cancer models and human head and neck cell model (HN30) (Liu et al., 2013). PORCN inhibitors LGK974, ETC-159, CGX1321, and RXC004 have reached Phase I clinical trials as treatment for various cancers (Shah et al., 2021). In addition to developmental cancers, Wnt signaling is also implicated in focal dermal hypoplasia (FDH). Specifically, this disease is characterized by mutations to PORCN itself that impact Wnt acylation and subsequent biological activity (Wang et al., 2007; Barrott et al., 2011).

#### 1.3.9 GOAT

GOAT is a key enzyme in the ghrelin signaling pathway. Ghrelin signaling was first linked to growth hormone secretion and appetite regulation (Müller et al., 2015). In addition it has implications in glucose metabolism, energy homeostasis, and organismal response to starvation (Egido et al., 2002; Reimer et al., 2003; Zhao et al., 2010a; Zhao et al., 2010b; Tong et al., 2010; Goldstein et al., 2011; Heppner et al., 2012; Li et al., 2012; Yada et al., 2014; Gagnon et al., 2015). Less obviously ghrelin signaling has also been implicated in cardio-protection, protection against muscle atrophy, and bone metabolism (Nass et al., 2008; Müller et al., 2015; Pearson et al., 2019; Tokudome and Kangawa, 2019; Agosti et al., 2020; Wu et al., 2020). Most recently ghrelin acylation and signaling has been linked to addictive behavior and alcoholism (Zallar et al., 2017; Farokhnia et al., 2019; Farokhnia et al., 2020; Farokhnia et al., 2021). Several classes of GOAT inhibitors that have been developed. The first are peptide-based drugs that mimic the product and/or substrate of GOAT. These molecules tend to be potent inhibitors, but have received little pharmaceutical interest due to their likely limited oral bioavailability (Iyer et al., 2020; Moose et al., 2020). Amongst small-molecule GOAT inhibitors, LY3073084 is in clinical trials for treatment of several metabolism-related disorders and BI 1356225 has been investigated in Phase 1 trials for treatment of obesity (Bianzano et al., 2023). Several more small molecule GOAT inhibitors have been reported, some with picomolar IC_50_, but these have yet to reach clinical trials (Moose et al., 2020).

## 2 Modeling MBOAT structure using computational methods

Like many integral membrane proteins, MBOATs have proven to be challenging to solubilize and purify for functional and structural studies. While recent work has accomplished significant advances in experimental structural determination of MBOAT family members, several MBOAT structural models were created using computational methods. These computational models proved useful for interpreting and designing biochemical studies of these enzymes, and comparison to more recently released structures of MBOAT family members demonstrated the power of computational methods to generate reasonable models for MBOATs.

### 2.1 GOAT

**FIGURE 4:**
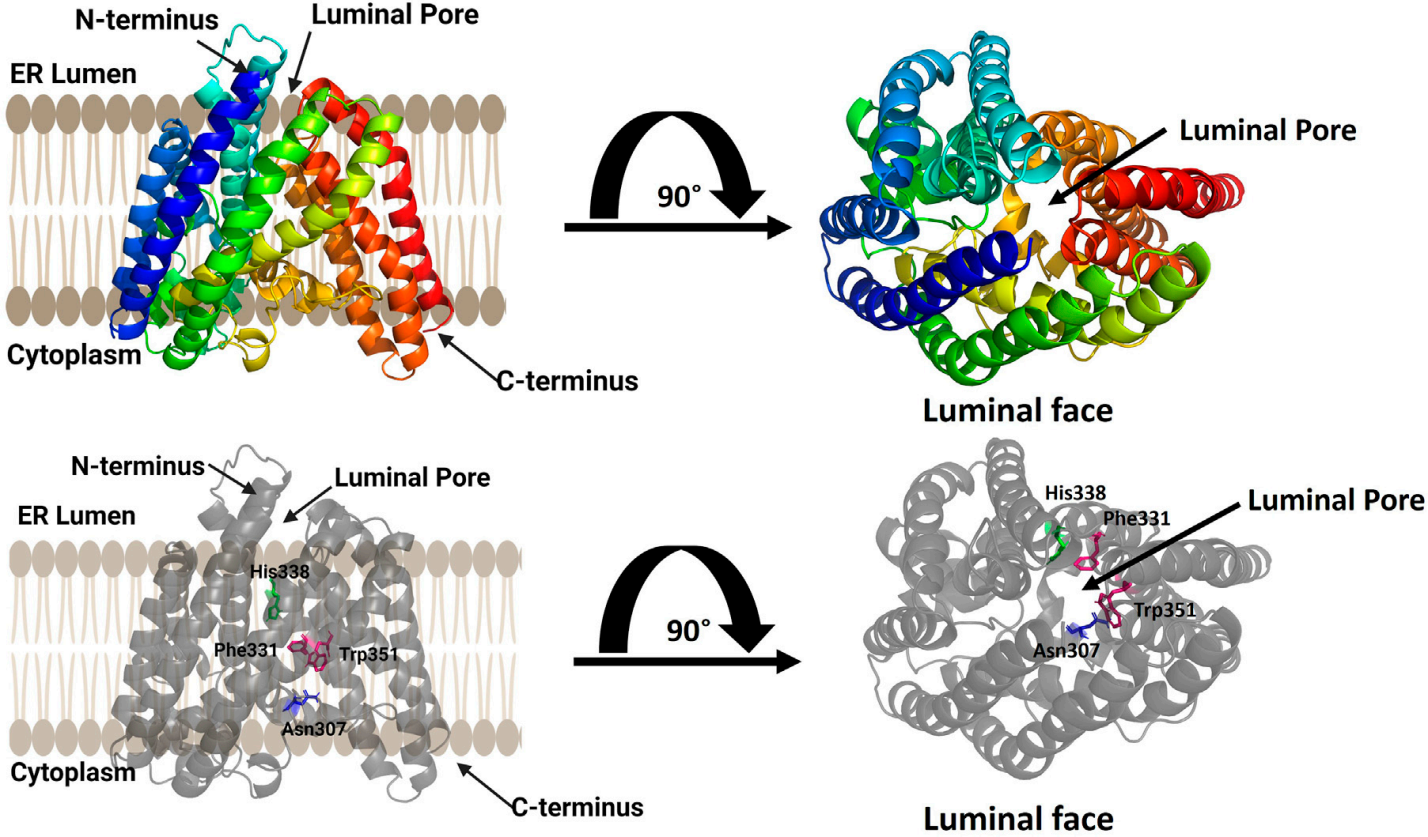
Computational model of ghrelin O-acyltransferase (GOAT). GOAT has 11 transmembrane helices and a transmembrane catalytic channel containing amino acids required for activity such as His 338 (green) and Asn 307 (blue) and residues implicated in acyl donor selectivity such as Trp351 and Phe331 (purple). The luminal pore for ghrelin binding and the catalytic channel can be seen in the top view from the luminal face of the membrane (right). Figure created using Biorender.

GOAT was computationally modeled using coevolutionary contact analysis combined with atomistic molecular dynamics (Figure 4) (Campaña et al., 2019). Coevolutionary contacts analysis relies on the hypothesis that amino acids that contact each other within a folded protein will co-evolve to maintain their interaction to create the most energetically favorable fold (Marks et al., 2012; Ovchinnikov et al., 2017). Using multiple sequence alignments to identify probable coevolutionary contacts, Campana and co-workers developed a set of distance constraints for computationally modeling human GOAT using standard protein folding approaches (Campaña et al., 2019). An array of 30,000 potential structures were created and evaluated for agreement with coevolutionary contact and topological constraints, leading to a best-fit structural model. This model was then embedded in a virtual lipid membrane and energy minimized by molecular dynamics (Campaña et al., 2019).

The structural model of human GOAT oriented the N-terminus within the ER lumen and the C-terminus in the cytoplasm and contained 11 transmembrane, consistent with a previously published topology of mouse GOAT (Taylor et al., 2013). Surprisingly, the GOAT model contains a channel through the core the enzyme connecting the lumen to the cytoplasm. This channel contains the conserved catalytically essential His338 residue, and extensive mutagenesis confirmed the functional requirement of this channel (Campaña et al., 2019). This channel and the core transmembrane helices surrounding it form what is now considered the MBOAT central core fold as denoted by Ma and co-workers (Ma et al., 2018).

The acyl donor octanoyl-CoA was docked into the GOAT model, illustrating the coenzyme A binding site on the cytosolic exposed face of the enzyme. The acyl donor chain penetrates into the core of GOAT and makes a turn to position it favorably for interactions with His338 and Asn307 (Campaña et al., 2019). Upon alanine mutagenesis of two aromatic residues contacting the distal end of the octanoyl chain, Trp351 and Phe331, GOAT lost the substrate preference for octanoyl-CoA and preferred longer-chain fatty acid chains as acyl donors (Campaña et al., 2019). This GOAT model and associated biochemical studies illustrated the structural basis for the unique octanoyl acyl chain selectivity exhibited by this enzyme in modifying ghrelin (Kojima et al., 1999; Darling et al., 2015), and is particularly important as there are no reported crystal or cryo-EM structures of GOAT.

### 2.2 HHAT

An HHAT homology model was published in 2021 by Lanyon-Hogg and co-workers. The published crystal structure of DltB and two published topology models for HHAT proved sufficient to support construction of this homology model (Konitsiotis et al., 2015; Matevossian and Resh, 2015; Ma et al., 2018). This model contained the protein/peptide MBOAT central core containing the catalytically essential His379 and Asp339 residues (Lanyon-Hogg et al., 2021). In addition, residues Pro212, Val213, and His215 involved in binding an HHAT inhibitor, (+)-6 IMP-1575, also located in this central core. The model has 10 integral membrane helices, with the N-terminus in the cytosol and the C-terminus in the luminal (Lanyon-Hogg et al., 2021). The overall shape of the HHAT model is consistent with the tent-like structure of the other protein/peptide acylating MBOATs. This model tends to be overlooked as experimental structures of HHAT were also published in 2021 as described below.

### 2.3 PORCN

Two computationally-derived structural models for PORCN were published in 2021. One model by Galli and co-workers was developed using homology modeling coupled with partial permeabilization studies and N-linked glycosylation analysis to establish the PORCN membrane topology (Galli et al., 2021). In this study, six different algorithms were applied to predict the PORCN membrane topology which yielded a range of 8–11 transmembrane domains. To experimentally determine the PORCN topology, introduction of antibody epitopes and N-linked glycosylation sites were used to identify if those epitopes/sites were exposed to the luminal space or the cytosol. Guided by these combined analyses, a homology model was developed consisting of nine transmembrane domains and two reentrant loops with the N-terminus facing the lumen and C-terminus in the cytosol. This model contains a funnel on the luminal side of the structure leading to a transmembrane tunnel, with the conserved His341 residue located in the center of the funnel (Galli et al., 2021).

A second PORCN computational model was created using homology modeling guided by the published MBOAT structures available at the time (Yu et al., 2021). Yu and co-workers used multiple sequence analysis algorithms and MODELLER to create their homology model for PORCN, which has ten transmembrane domains and both the N-terminus and C-terminus located in the cytoplasm. This model also contains a transmembrane tunnel with the conserved His341 residue and depicts binding sites for both the acyl donor and Wnt substrates. Several PORCN inhibitors were also docked into the homology model, with these inhibitors binding into the enzyme active site. These two PORCN structural models provided important context for understanding how this enzyme binds its substrates and catalyzes Wnt acylation, and served as points for comparison for the experimentally determined structure of PORCN released the following year, as described below (Liu et al., 2022).

## 3 Experimentally determined MBOAT structures

### 3.1 DltB

In 2018, Ma and co-workers published the crystallographic structure of bacterial *D*-alanyltransferase DltB, the first such structure of an MBOAT family member (Ma et al., 2018). DltB is essential for the D-alanylation of cell wall teichoic acids, using an acyl carrier protein DltC as the acyl donor (Ma et al., 2018). The structure of this bacterial MBOAT homolog facilitated homology modeling of PORCN and provided a valuable reference to validate features of the computational GOAT structural model (Campaña et al., 2019; Galli et al., 2021; Yu et al., 2021). DltB was expressed in bacteria and solubilized with n-decyl-β-D-maltopyranoside, with samples for crystallization solubilized in n-decyl-nonyl-β-D-glucopyranoside (Ma et al., 2018). The DltB structure contains 17 helices and both the N-terminus and C-terminus are on the same side of the membrane. Of the 17 helical domains, 11 are transmembrane domains that form a ring-shaped cone with a conserved MBOAT structural core and a transmembrane channel with a funnel on the extracellular interface. At the time of publication, the DltB structure was described as a funnel with a fold dissimilar to any available structures. As described below, subsequent structures of additional MBOATs have revealed that DltB contains most of the conserved features of this enzyme family.

### 3.2 hACAT/hSOAT

2020 brought the MBOAT community the first structures of mammalian MBOATs, with two ACAT1/SOAT1 structures and two DGAT1 structures published in the same issue of Nature followed shortly by a third ACAT1/SOAT1 (Guan et al., 2020; Long et al., 2020; Qian et al., 2020; Sui et al., 2020; Wang et al., 2020).

#### 3.2.1 ACAT1/SOAT1

**FIGURE 5:**
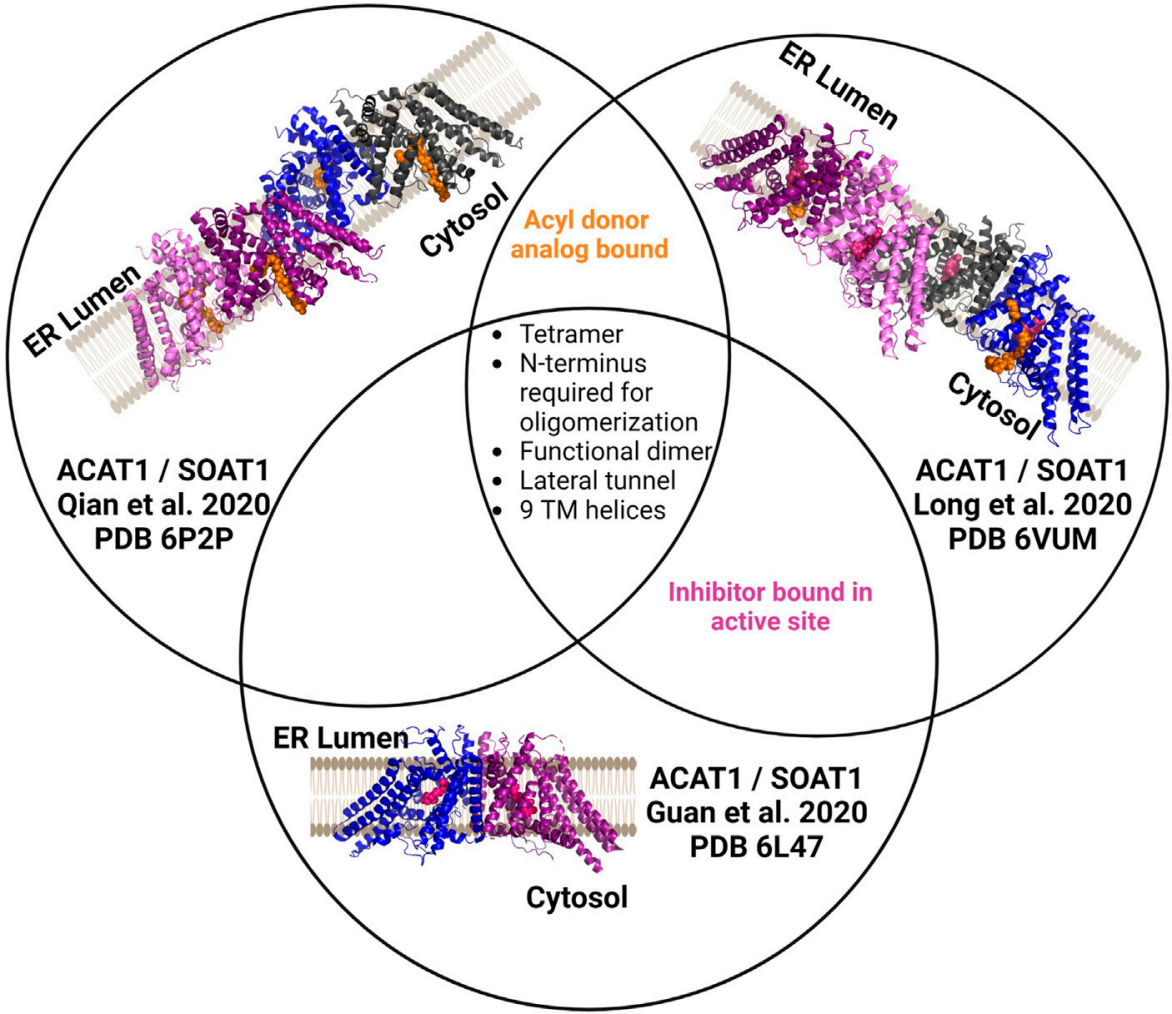
Independently determined structures of ACAT1/SOAT1 reveal common characteristics. All three published ACAT1/SOAT1 structures are consistent in identifying a multimeric complex, with at least two structures of the three also containing bound acyl donor and/or an inhibitor bound in the catalytic core of the enzyme. Different colors denote each monomeric unit within the dimeric or tetrameric complexes. Bound inhibitors are shown in purple, and acyl donor analogs are in orange. Figure created using Biorender.

ACAT1/SOAT1 was purified as a tetrameric protein or dimer of dimers in the three published structures (Figure 5) (Guan et al., 2020; Long et al., 2020; Qian et al., 2020). The four monomers in the ACAT1/SOAT1 complex provide sufficient size and mass to allow cryo-EM analysis of the enzyme complex alone. Each ACAT1/SOAT1 monomer contains nine transmembrane helices, where the N-terminus “hugs” or folds into the other N-terminii of the other monomers forming the tetrameric complex (Guan et al., 2020; Long et al., 2020; Qian et al., 2020). The tetramer, dimer, and monomer were tested for catalytic activity and it was found that only the monomer lacked ACAT1/SOAT1 acylation activity consistent with the dimer forming the catalytically active unit (Guan et al., 2020; Qian et al., 2020).

In the structures by Long et al. and Qian et al., several amino acids (His425, Tyr433, Lys445, Ser456) interact with acyl-CoA and were annotated to either form the cytosolic interface or a cytosolic tunnel where the acyl donor binds (Long et al., 2020; Qian et al., 2020). However, Guan and co-workers did not identify an oleoyl-CoA binding site as the other structures described. These authors hypothesize that the inhibitor CI-976 bound in their preparation leads to an ACAT1/SOAT1 conformation that does not effectively bind the acyl-CoA donor.

In addition to the residues noted above in the acyl donor binding site, Asn 421 was also shown to be required for ACAT1/SOAT1 acylation activity (Long et al., 2020; Qian et al., 2020). These residues all reside in the catalytic core and have contacts with acyl-CoA. The structures also reveal two cholesterol binding sites, with one serving as the substrate binding site for this cholesterol acyltransferase and the other proposed to serve an allosteric role (Long et al., 2020). Qian et al. and Guan et al. suggest evidence of not only a CoA substrate tunnel, but a transmembrane channel that is hypothesized to bind cholesterol (Guan et al., 2020; Qian et al., 2020). This channel converges with the acyl-CoA donor binding site/tunnel at the conserved His460 residues within the enzyme core. Mutations along this tunnel were detrimental to activity, consistent with the proposed role for this feature in acylation catalysis (Qian et al., 2020).

#### 3.2.2 ACAT2/SOAT2

**FIGURE 6:**
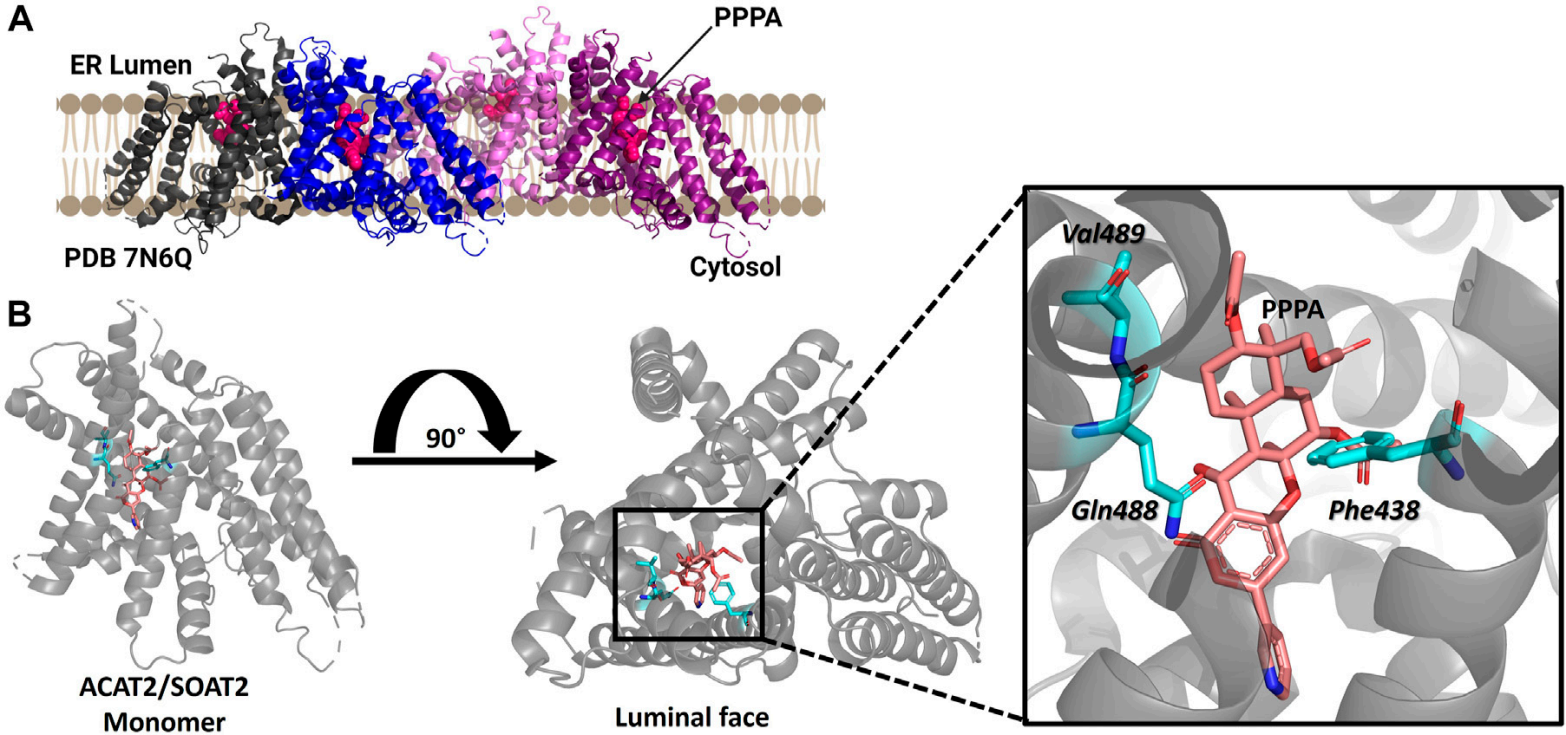
Structure of ACAT2/SOAT2 bound to the PPPA inhibitor. **(A)** ACAT2/SOAT2 forms a tetramer, but only requires dimer formation for acylation activity. Monomers are shown in gray, blue, pink, and purple, and the PPPA inhibitor in each monomer is hot pink. **(B)** One molecule of PPPA binds per monomer of ACAT2/SOAT2, contacting residues Val489, Gln488, and Phe438. PDB ID: 7N6Q. Figure created using Biorender.

ACAT2/SOAT2 was purified as a dimer of dimers with each monomer containing nine transmembrane helices with only a RMSD of 0.8Å between ACAT1/SOAT1 and ACAT2/SOAT2 (Figure 6) (Long et al., 2021). ACAT2/SOAT2 also contains the conserved acyl-CoA binding pocket and the hydrophobic core for cholesterol esterification observed in ACAT1/SOAT1 (Guan et al., 2020; Long et al., 2020; Qian et al., 2020; Long et al., 2021; Long et al., 2022). The cholesterol tunnel described for ACAT1/SOAT1 is also present in ACAT2/SOAT2 and has access to the proposed catalytic histidine His434 (Long et al., 2021). ACAT2/SOAT2 also contained an additional cholesterol binding site each monomer separate from the cholesterol present in the substrate binding site within the catalytic core. In both ACAT1/SOAT1 and ACAT2/SOAT2 the second cholesterol binding site is proposed to be allosteric and mutation of both of cholesterol binding sites reduce acylation activity (Long et al., 2020; Long et al., 2021).

### 3.3 hDGAT1

**FIGURE 7:**
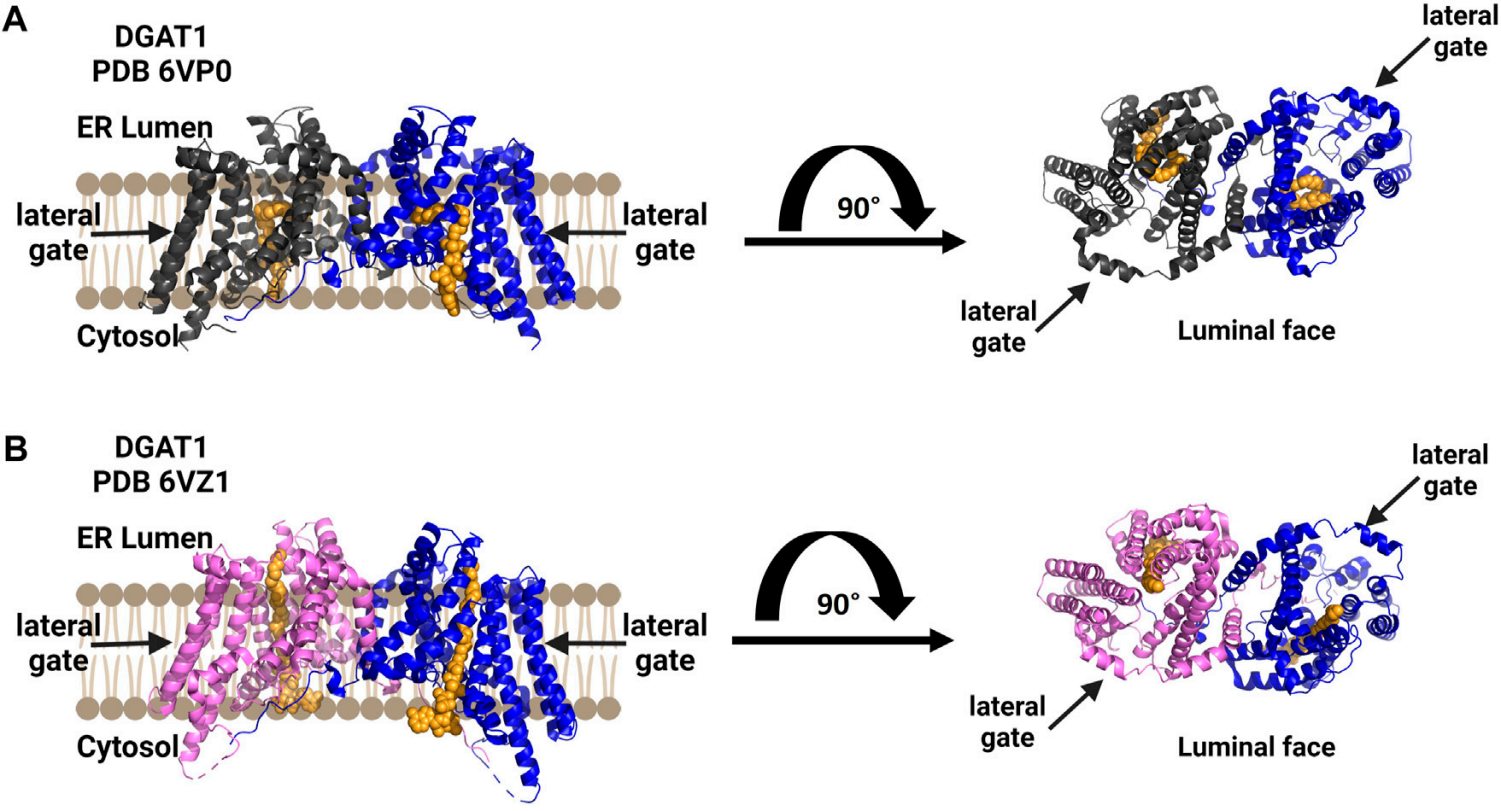
DGAT1 is a dimeric enzyme with a central cavity and a lateral gate. Independent structures confirm the formation of functional dimers (blue and dark gray), with a lateral gate for substrate access denoted by black arrows on each structure’s top view from the luminal face (right). Both structures contain a non-hydrolyzable CoA analog in orange. **(A)** Structure by Wang and co-workers (PDB ID 6VP0). **(B)** Structure by Sui and co-workers (PDB ID 6VZ1). Figure created using Biorender.

Human diacylglycerol acyltransferase I (hDGAT1) was resolved as a dimer and structurally analyzed by cryo-EM (Figure 7) (Sui et al., 2020; Wang et al., 2020). In the study by Sui and co-workers, the amphipol PMAL-C8 was used to maintain a homogenous oligomer structure for structure determination (Sui et al., 2020). hDGAT1 has nine transmembrane domains with the N-terminus facing the cytosol and the C-terminus facing the lumen (Sui et al., 2020; Wang et al., 2020). The hDGAT1 dimer is formed through hydrogen-bonding and interactions with phospholipids present between monomers. DGAT1 has a large central core and a lateral gate that is open to the membrane. Its substrate diacylglycerol (DAG) can enter the membrane from the lumen or the cytosol. The conserved histidine His415 is also present in the large central core where the lateral gate connects to the active site (Sui et al., 2020; Wang et al., 2020) Oleoly-CoA binds within the central core, but the acyl binding site is long enough to accommodate longer chain fatty acids (Sui et al., 2020). Similar to the cholesterol acyltransferases, DGAT1 has a channel connecting to the lipid membrane and Sui and co-workers identified a diacylglycerol in this channel that co-purified with the enzyme. Wang et al. also found a similar lateral gateway, but could not resolve the density present in this chamber (Wang et al., 2020). DGAT has a bend in the lateral gateway that is proposed to select lipid substrates over rigid like structures like cholesterol (Sui et al., 2020). For a more detailed comparison of these structures, the authors direct attention to a recent review of DGAT enzymes (Chen et al., 2022).

### 3.4 cLPCAT3

**FIGURE 8:**
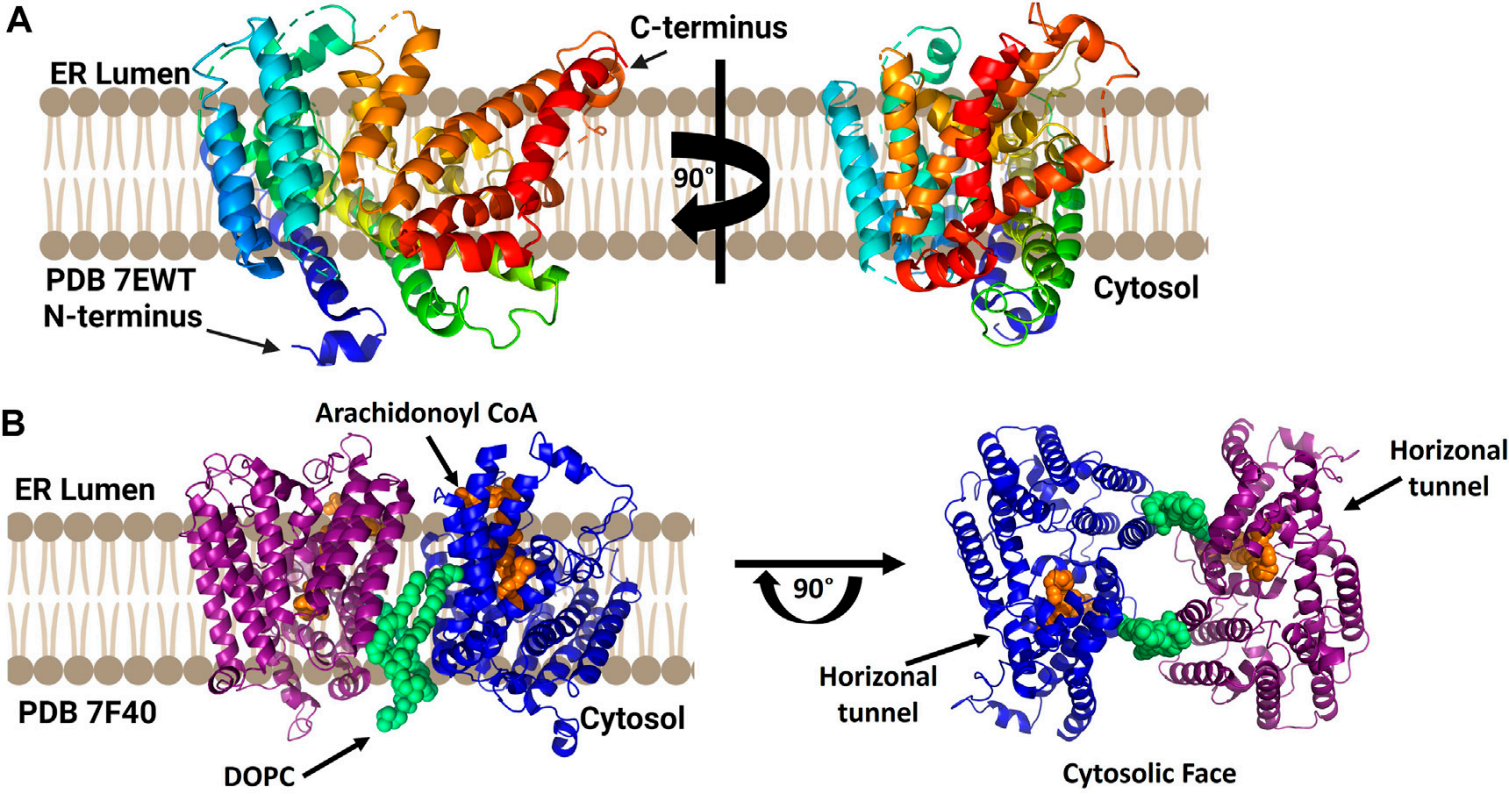
cLPCAT3 structural analysis reveals a condition-dependent dimerization state. **(A)** X-ray crystal structure of cLPCAT3 (PDB ID 7EWT) solved without substrates bound. For comparison to the dimer structure from cryoelectron microscopy, the rotated image on the right is presented in the same orientation as the right (blue) subunit of the dimer in left side of panel **(B)** Dimeric structure of cLPCAT3 solved by cryoelectron microscopy (PDB ID 7F40) with arachidonoyl CoA (orange) and 1,2-dioleoyl-sn-glycero-3-phosphocholine (DOPC, lime green) bound to each monomer. Arrows on the cytosolic top view indicate the lateral/horizontal tunnel for acyl acceptor entry. Figure created using Biorender.

While a mammalian lysophosphatidylcholine acyltransferase structure remains to be reported, Zhang and co-workers reported the crystal structure of chicken lysophophatidylcholine acyltransferase 3 (LPCAT3) (Figure 8). To obtain this structure, LPCAT3 was solubilized in undecyl maltoside and two residues at the enzyme C-terminus were removed to improve homogeneity and stability resulting structures at 3.4 Å resolution (Zhang et al., 2021). LPCAT3 has an overall bell shape comprising 11 transmembrane domains and 6 short helices, with the transmembrane domains surrounding a central cavity (Zhang et al., 2021). Similar to ACAT1/SOAT1 and DGAT1, LPCAT3 also contains a lateral tunnel connecting the central cavity to the surrounding membrane. To characterize the substrate binding pockets in LPCAT3, cryo-EM was utilized as substrate-bound crystals were not obtained. When purified in LMNG for cryo-EM analysis, cLPCAT3 was oligomeric. The cryo-EM structure was solved in the presence of both enzyme substrates, the arachidonoyl-CoA (araCoA) acyl donor and 1-dodecanoyl-sn-glyero-3-phosphocholine (12:0-LPC) in a dimeric state (Zhang et al., 2021). The cLPCAT3/araCoA structure had an araCoA in the central cavity with the acyl chain downstream of the unsaturation “kink” entering a side pocket. This acyl chain conformation leaves the horizonal tunnel available for the LPCAT3 acyl acceptor, resulting in araCoA lining up with the conserved His388 residue and acyl acceptor within the enzyme core (Zhang et al., 2021). Cross-linking studies support the biological relevance of cLPCAT3 dimerization, but the specific function of monomer-monomer interaction remains to be understood (Zhang et al., 2021). Looking towards the catalytic mechanism for lysophospholipid acylation, the authors suggest a mechanism for acyl transfer wherein the acyl donor and acyl acceptor are bound simultaneously within the enzyme core. The carbonyl carbon of the acyl donor is activated by Asn352 in a manner reminiscent of oxyanion hole interactions in serine proteases, while the sn-2 hydroxyl of the acyl receiver is activated by His388 acting as a general base. Acylation proceeds by attack of the activated sn-2 hydroxyl group on the activate thioester of the acyl donor and resolves with transfer of the acyl chain to the acceptor (Zhang et al., 2021).

### 3.5 hHHAT

**FIGURE 9:**
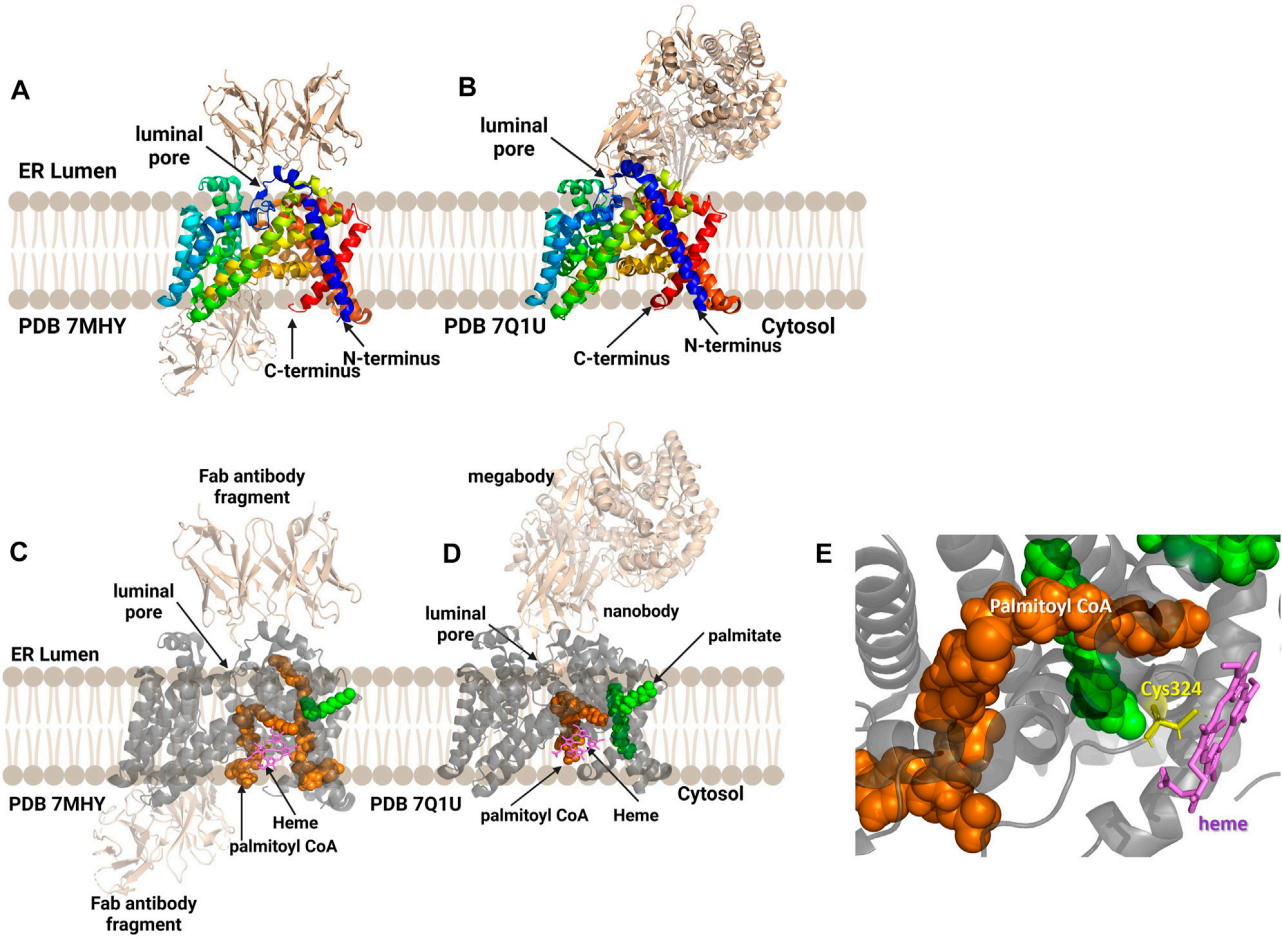
Antibody-based complexes enabled cryoelectron microscopy determination of HHAT structure, revealing substrate binding sites, inhibitor binding, and an unanticipated heme cofactor. **(A)** Cryo-EM structure of HHAT by Jiang and co-workers (PDB ID 7MHY) shows the overall structure of the enzyme complexed to antibodies on both the cytoplasmic and luminal interfaces. **(B)** Binding to a designed megabody partner enabled the cryo-EM structure of HHAT by Coupland and co-workers (PDB ID 7Q1U). **(C, D)** Both groups report the presence of a heme (violet) in their structures; antibodies/megabody shown in beige, palmitoyl-CoA in orange, and palmitic acid in green. **(E)** Heme binding site in PDB ID 7Q1U showing heme iron coordination by Cys324. Figure created using Biorender.

Human hedgehog acyltransferase (hHHAT) is a monomeric enzyme whose size is insufficient to support cryo-EM analysis of the enzyme alone, leading two research groups to employ antibody-derived binding partners to increase the enzyme-complex size sufficiently to allow structure determination (Figure 9) (Coupland et al., 2021; Jiang et al., 2021). The HHAT structures reveal 12 transmembrane helices connected by intervening alpha helical and loop regions forming the now-canonical “MBOAT fold”. This aligns the conserved His379 residue with the conserved residues in the structures of other MBOAT family members (Coupland et al., 2021; Jiang et al., 2021). Jiang and co-workers modeled a palmitoyl-CoA acyl donor into the central cavity of the enzyme. The central cavity connects to the cytosolic face of the ER membrane (Jiang et al., 2021). Coupland et al. also resolved a non-hydrolyzable palm-CoA analog bound within the central tunnel which would span from the cytosolic to luminal sides of the ER membrane. This places the palmitoyl-CoA close to the conserved His379 and catalytically required Asp339 residues (Coupland et al., 2021). Surprisingly, both structures revealed a heme coordinated to Cys324, with subsequent mutagenesis and functional studies indicating this heme is required for enzyme stability (Coupland et al., 2021; Jiang et al., 2021). Jiang and co-workers suggest a one-step mechanism where Asp339 activates the N-terminal cysteine of Hedgehog for nucleophilic attack on the thioester carbonyl atom of the acyl donor, with the Oxford group supporting further studies before defining a mechanism (Coupland et al., 2021; Jiang et al., 2021). The HHAT structure was solved with the IMP1575 inhibitor bound by three labs (Coupland et al., 2021; Jiang et al., 2021; Liu et al., 2022). IMP1575 is the most potent reported HHAT inhibitor (Lanyon-Hogg et al., 2021). This structure depicted a directed binding interaction to the catalytic His379 and creates a conformational change within the enzyme. This conformational change rearranges Asp339, Asn443, and Trp335. Trp335 rotates into the binding pocket, and this residue is responsible for preventing water penetration into the enzyme core without palmitoyl-CoA bound (Coupland et al., 2021).

### 3.6 hPORCN

**FIGURE 10:**
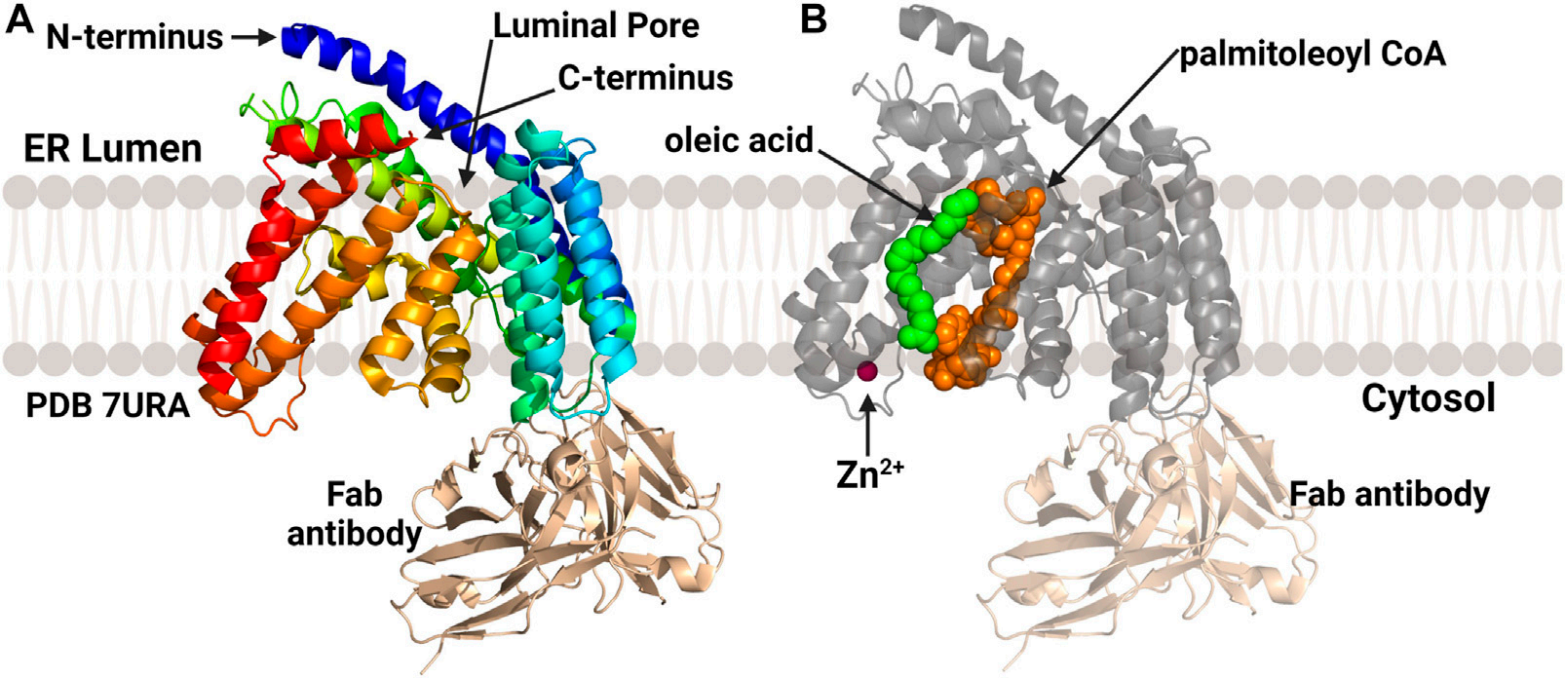
Structures of PORCN bound to substrates. **(A)** Overall structure of PORCN with 11 transmembrane domains and both the N-terminus and C-terminus in the ER lumen. **(B)** PORCN in complex with palmitoyl-CoA (orange) (PDB ID 7URA), showing the bound zinc ion (magenta) and palmitic acid (green). The antibody structural chaperone is shown in wheat. Figure created using Biorender.

In 2022 the structure of PORCN, the acyltransferase responsible for Wnt acylation (Figure 10), was published by Lie and co-workers (Liu et al., 2022). Similar to HHAT, PORCN was purified as a monomer which required antibody-derived binding partners to reach the mass/size range compatible with cryo-EM methods. Consistent with the computational models described above, PORCN was determined to contain 11 transmembrane helical domains with 6 intervening alpha helices and 2 beta strands. The Wnt substrate binding site on the luminal face of the enzyme is composed of portions of TMs 1, 2, 5, and 7. The catalytic core and central domain of PORCN containing the conserved His336 residue are consistent with other protein/peptide acylating MBOAT. A co-structure with palmitoleoyl-CoA showed this core also binds the acyl donor substrate in a cavity made by TM7 and TM10. A zinc ion found coordinated to four residues (Cys370, Cys376, Cys380, and His382) consistent with previous studies of PORCN, although the role of zinc in PORCN structure and function remains to be defined (Lee et al., 2019; Liu et al., 2022). In addition to the palmitoleoyl-CoA co-structure, one additional co-structure was solved with a PORCN inhibitor LGK974 bound (Liu et al., 2013). This co-structure found that Ser332 of PORCN interacts with the carbonyl oxygen of LGK974 and is consistent with the acyl-CoA competitive nature of this inhibitor (Liu et al., 2022).

## 4 Conclusion and lessons learned from MBOAT structures

For more than two decades, the MBOAT enzyme family has posed intriguing challenges across the fields of enzymology, structural biology, lipid synthesis and remodeling, and cellular signaling by acylated proteins (Hofmann, 2000). The recent explosion in MBOAT structures and structural models spanning all three classes of acylation substrates has dramatically advanced our understanding of these integral membrane enzymes (Table 1). These structures have revealed family-wide shared characteristics, illustrated distinct properties of enzymes depending on their acyl acceptor substrates, and brought into clearer focus questions regarding their acylation mechanisms and the potential for inhibitor creation towards therapeutic application. Between experimental structural studies, structure modeling using the approaches described in this review, and application of AI-based folding prediction approaches such as AlphaFold (Jumper et al., 2021; Binder et al., 2022), the prospects for continuing the rapid advancement in our understanding of the MBOAT enzyme family are incredibly promising.

**TABLE 1:** MBOAT structures from computational and experimental studies.

MBOAT	Year	Monomer/Multimer	PDB ID(s)	Reference
	Reported			
*Computational Modeling*				
GOAT	2019	Monomer	n/a	Campaña et al. (2019)
PORCN	2020	Monomer	n/a	Galli et al. (2021)
PORCN	2020	Monomer	n/a	Yu et al. (2021)
*X-ray crystallography*				
DltB	2018	Monomer	6BUG, 6BUH,	Ma et al. (2018)
			6BUI	
LPCAT3	2021	Monomer	7EWT	Zhang et al. (2021)
*Cryoelectron microscopy*				
ACAT1/SOAT1	2020	Tetramer	6VUM	Long et al. (2020)
ACAT1/SOAT1	2020	Tetramer	6P2J, 6P2P	Qian et al. (2020)
ACAT1/SOAT1	2020	Tetramer	6L47, 6L48	Guan et al. (2020)
DGAT1	2020	Dimer	6VYI, 6VZ1	Sui et al. (2020)
DGAT1	2020	Dimer	6VP0	Wang et al. (2020)
ACAT2/SOAT2	2021	Tetramer	7N6R, 7N6Q	Long et al. (2021)
LPCAT3	2021	Dimer	7F3X, 7F40	Zhang et al. (2021)
HHAT	2021	Monomer	7Q1U, 7Q6Z	Coupland et al. (2021)
HHAT	2021	Monomer	7MHY	Jiang et al. (2021)
HHAT	2022	Monomer	7URF	Liu et al. (2022)
PORCN	2022	Monomer	7URA, 7URC,	Liu et al. (2022)
			7URD,7URE	

Comparison of all reported MBOAT structures supports the establishment of a “MBOAT core” fold, with a cone shaped bundle of transmembrane domains surrounding a conserved core region. This core region, comprising helices and domains annotated as cytoplasmic loops in topological studies, contains an open channel or cavity within which sits the conserved histidine residues that is a hallmark of MBOAT family members. This central catalytic channel also connects to an acyl donor binding site exposed to the cytoplasmic space. We note that, except for the bacterial MBOAT DltB, all other MBOATS utilize acyl-coenzyme A as their acyl donor substrates. These enzymes feature catalytic cores that lie within the plane of their surrounding membranes, presenting an elegant answer to early questions in the MBOAT field of whether their active sites would lie on the cytoplasmic or luminal/extracellular faces of these acyltransferases.

Within the MBOAT family, structural and mechanistic distinctions are most notable between the protein-modifying members (GOAT, HHAT, and PORCN) and the small molecule/lipid modifying enzymes. The protein-modifying members are active as monomers, which corresponds with their experimentally solved structures. In contrast, the small molecule/lipid acylation MBOATs function as dimers or higher oligomers consistent with the tetramers or dimer of dimers in the experimentally determined structures. Focusing on the location of the acyl acceptor entry site and nature of the catalytic channel, the protein-modifying family members all contain channels that completely span the membrane with acyl acceptors (ghrelin, Hedgehog, and Wnt) entering the enzyme through a luminal pore and acyl donors binding from the cytoplasmic interface. This catalytic topology matches what would be expected for modification of proteins transiting the secretion pathway through the ER lumen. The channel in the protein/peptide MBOATs structures explains how substrates on opposites sides of the membrane interact to effect substrate acylation, a long-outstanding question in the MBOAT community. Rather than entry from the lumen, acyl acceptors for the small molecule/lipid modifying family members enter through a “lateral gate” into the central channel/core that presumably allows these hydrophobic substrates to transit from the membrane bilayer into the enzyme active site for acylation. Several of the small molecule/lipid modifying MBOATs also contain secondary binding sites for cholesterol and lipids, although the functional importance of these sites remains to be conclusively demonstrated. Perhaps the most surprising finding amongst the MBOAT structures is the heme binding site within HHAT that impacts enzyme stability. It is hypothesized the heme binding is essential to stabilize enzyme structure, rather than participating directly in the enzyme catalysis of substrate acylation.

Moving forward with this newfound bounty of MBOAT structural data, our studies should focus on a comprehensive mechanistic understanding of acyl transfer by MBOATs and development of potent and specific MBOAT inhibitors. For example, on the mechanistic front it remains unresolved whether these enzymes use a one-step direct transfer mechanism with the acyl chain moving directly to acyl acceptor or a two-step transfer mechanism involving an acyl-enzyme intermediate. Given the structural and topological distinctions between the protein- and small molecule/lipid-modifying MBOAT family members, it will be interesting to determine whether these distinct enzyme subclasses also exhibit mechanistic differences. Combination of current and future structural information with mechanistic insights will guide the creation of the next-generation of MBOAT inhibitors, which are needed to explore and exploit the therapeutic potential of these enzymes for treating a range of human diseases.
